# Chronic hexavalent chromium exposure induces oxidative stress-mediated molecular cascades in Thymallus grubii gills: evidence from integrated transcriptomics and metabolomics

**DOI:** 10.3389/fimmu.2025.1633174

**Published:** 2025-07-09

**Authors:** Xinchi Shang, Xinghua Che, Kai Ma, Bo Ma, Huizhi Sun, Wenhua Wu, Hailong He, Meiqi Xing, Wei Xu, Yongquan Zhang

**Affiliations:** ^1^ Key Open Laboratory of Cold Water Fish Germplasm Resources and Breeding of Heilongjiang Province, Heilongjiang River Fisheries Research Institute, Chinese Academy of Fishery Sciences, Harbin, China; ^2^ College of Life Science, Northeast Agricultural University, Harbin, China; ^3^ College of Marine Biology and Fisheries, Hainan University, Haikou, China; ^4^ Heilongjiang Aquatic Animal Resource Conservation Center, Harbin, Heilongjiang, China

**Keywords:** gill, metabolome, transcriptome, Cr(VI) stress, inflammatory responses

## Abstract

**Introduction:**

Cr(VI) is a heavy metal contaminant, can diffuse to ecosystems and harm aquatic animals. Gills, as a vital organ in direct contact with the aquatic environment, have become a key target tissue for assessing the toxicological effects of heavy metal pollution of water bodies due to their sensitivity to heavy metal exposure. However, 3the effects of Cr(VI) on the gill tissues in fish have been less studied. In this study, we revealed the multiple effects of chromium toxicity by assessing the oxidative damage, transcriptomic and metabolomic changes of Cr(VI) on gill tissues of *Thymallus grubii*.

**Methods:**

A total of 270 fishes were stratified into three experimental groups: control group, low-concentration exposure group (0.2 mg/L), and high-concentration exposure group (1 mg/L). In this study, we revealed the multiple effects of chromium toxicity by assessing the oxidative damage, transcriptomic and metabolomic changes of Cr(VI) on gill tissues of *Thymallus grubii*.

**Results:**

Cr(VI) stress can lead to gill damage with significant reduction in gill filament thickness, significant thinning of gill lamellae, and congestion of epithelial blood vessels. Cr(VI) stress significant increases in H_2_O_2_ and MDA levels and significant decreases in antioxidant enzyme activity levels (SOD, GSH-Px, and T-AOC) and energy metabolism-related ATPase activity levels (Na^+^K^+^-ATPase, Ca^2+^-ATPase, and Mg^2+^-ATPase). Cr(VI) stress induced disturbances in gill arachidonic acid metabolism leading to the release of pro-inflammatory metabolites (e.g., thromboxane A2 and prostaglandin J2) accompanied by the accumulation of oxidised glutathione. However, the synthesis of metabolites with anti-inflammatory/antioxidant functions (e.g. GABA, quinidine and l-artitic acid) was reduced. Transcriptomics and metabolomic coanalyses revealed that Cr(VI) induced *PPAR-γ* inactivation to deregulate *COX-2*, which disrupted arachidonic acid metabolic pathways, leading to oxidative stress, apoptosis, and release of inflammatory factors. Disorders of arachidonic acid metabolism led to the release of proinflammatory metabolites (such as thromboxane A2 and prostaglandin J2), and decreased levels of reduced glutathione.

**Discussion:**

The effects of Cr(VI) exposure on gill gene expression and metabolism were analysed using RT-PCR, transcriptomic, and metabolomic approaches. In summary, we better understand the toxic effects of Cr(VI) on gill tissues of aquatic animals. Targeted activation of *PPAR-γ* and supplementation with anti-inflammatory metabolites such as GABA, quinidine and l- artitic acid may be potential intervention strategies to reverse Cr(VI) toxicity.

## 1 Introduction

Global water resources are increasingly polluted by heavy metals due to rapid industrial development and agricultural activities, seriously threatening the aquatic animal health and disrupting the balance of ecosystems (1). As a redox-active transition metal ubiquitous in industrial systems, chromium persists in natural ecosystems through multiple valence states, of which Cr^3+^ and Cr(VI)(Cr^6+^) are common ionic forms (2). Cr^6+^ has strong oxidative activity and chemical toxicity and can cause allergic dermatitis, neurotoxicity, genotoxicity, and cancers (3). Cr^6+^ enters cells in the chromate ionic state, crossing cell membranes through nonspecific phosphate or sulfate anion carriers, leading to mitochondrial damage and cellular DNA damage (4). Owing to human activities, the Cr^6+^ released from industrial production migrates to groundwater by entering surface water and soil (5). Hexavalent chromium salts are more soluble than Cr^3+^; thus, Cr^6+^ is relatively more mobile. In aquatic environments, Cr^6+^ is present for longer periods of time and is more harmful to biological systems; therefore, Cr^6+^ is more toxic than Cr^3+^, which is considered one of the most harmful heavy metals (6). Fish is an important source of protein for humans, and enrichment of Cr^6+^ in contaminated fish tissue can be transferred to the human body and poses health risks. Therefore, the study of the effects of Cr^6+^ exposure on fish can provide a basis for the aquaculture industry to formulate aquatic product safety standards, and it is also an inevitable choice to safeguard human food safety and prevent and control environmental pollution.

Studies have shown that chronic exposure of animals to Cr^6+^ leads to oxidative stress and significantly increases the expression of apoptotic genes; moreover, Cr^6+^ can affect the normal function of mitochondria, transmembrane potential, and antioxidant enzyme activity, resulting in mitochondrial damage, which triggers a cascade of caspase proteases leading to apoptotic cell death (7–9). *In vitro* cytotoxicity experiments have shown that Cr^6+^ ions enter the cell through nonspecific ion channels in the cell membrane and generate large amounts of ROS during their reduction to the low-valent form of chromium and that the continuous accumulation of ROS in cells causes DNA and cellular damage (10, 11). Cr^6+^ exposure was found to cause neurotoxicity and induce oxidative damage in zebrafish (12). Gills are important organs for gas exchange, osmoregulation and detoxification in fish (13). Gills, characterized by substantial surface area and acute responsiveness to aquatic environmental fluctuations, serve as primary target sites for toxic metal accumulation in aquatic organisms (14). Several studies have confirmed that heavy metal exposure leads to gill damage in fish, with altered morphology and pathology, energy and metabolic imbalances, and impaired antioxidant systems (15–17).

Amur grayling (*Thymallus grubii*) is an important albino fish in China with high nutritional and economic values. *T. grubii* has become highly valuable as an economic species for cold Water aquaculture, possessing tasty meat, rich in unsaturated fatty acids, low cholesterol and high protein levels. However, due to environmental pollution and other anthropogenic disturbances, the population of *T. grubii* has declined sharply (18). In addition, understanding the response mechanism of *T. grubii* to heavy metal stress can help to provide scientific evidence for monitoring the ecological health of the watershed, and provide toxicological basis for the development of aquatic animal conservation strategies. In heavy metal exposure studies, transcriptomics analyses can comprehensively resolve the dynamics of gene expression in organisms under heavy metal stress. Metabolomics in heavy metal exposure studies can elucidate the details of metabolic processes that occur during environmental adaptation and provide a better understanding of these processes. However, the effects of Cr^6+^ stress on tissue, gill metabolism, and gene expression in *T. grubii* have not been reported. In the present study, we assessed the effects of chronic Cr^6+^ exposure on gill tissue structure, metabolism and gene expression in *T. grubii*. This study was to elucidate the effects of gill exposure to heavy metals on gene expression, transcriptomics and metabolomics to elucidate the contribution of Cr^6+^ to *T. grubii-*induced gill toxicity and the possible underlying mechanisms.

## 2 Materials and methods

### 2.1 Animals and diet

This study was approved by the Ethics Committee for Animal Experiments of the Heilongjiang Fisheries Research Institute, Chinese Academy of Fisheries Sciences (20241125–007). The Bohai Experimental Station of the Heilongjiang Provincial Fisheries Research Institute provided the fish for this experiment. Fish were temporarily reared in the aquarium for 30 days to acclimatize to the environment. A total of 270 fish of uniform size were selected and allocated to nine aquaria (three groups), with three biological replicates established in each group. Control fish tanks were filled with tap water. In the aquarium for the low-concentration exposure group (Lcr group: Cr^6+^ of 0.2 mg/L). In the aquarium for the high-concentration treatment group (Hcr group: Cr^6+^ of 1 mg/L). Cr^6+^ exposure levels refer to the results of previous studies and water resources ecosystem surveys (19–21). During the feeding experiment, the fish were handfed twice daily (at 8:30 a.m. and at 16:00 p.m.) to apparent satiation for 4 weeks. Experimental environment of fish: temperature, 10 ± 2°C; pH, 6.8 ± 0.44 dissolved oxygen, 5.57 ± 0.63 mg/L; ammonia concentration < 0.3 mg/L; nitrite concentration < 0.02 mg/L. Potassium dichromate solution was purchased from Merck, China (CAS: 7778-50-9, MDL: MFCD00011367, EC: 231-906-6). Each tank was replaced with 25% water daily.

### 2.2 Fish sampling

After 24 h of starvation, the fish were anaesthetized with 20.0 mg/L tricaine methanesulfonate (MS-222) at weeks 2 and 4 of the experiment. Eighteen fish per group were randomly selected and prepared for sampling. Fish were euthanized and gills, muscles, livers, and intestines were collected. The collected tissues were frozen in liquid nitrogen and transferred to a −80°C refrigerator.

### 2.3 Chromium analysis

Cr^6+^ levels in gills, liver, muscle, and intestines were only examined by the methods published in previous studies (22). First 0.1 g of sample, 5.0 mL of nitric acid was mixed in an ablative tube and 3.0 mL of ultrapure water was added. Mineralization of the samples was carried out in a MarXpress microwave ablation system (North Carolina, USA). Cooling 0.5 mL of internal standard was added and the sample was diluted to 50.0 mL with ddH_2_O and the experiment was repeated 3 times. Cr^6+^ levels in gills, liver, muscle, and intestines were quantified using an Agilent 7500cx ICP-MS (Agilent Technologies, Santa Clara, CA, USA). Cr^6+^ levels in water tanks were determined by the same method (**Table 1**).

**Table 1:** Cr^6+^ levels in water (mg/L).

Group	Nominal concentration	Actual Cr^6+^ concentration at 14 days	Actual Cr^6+^ concentration at 28 days
Control	0	0.004 ± 0.002	0.006 ± 0.001
Lcr group	0.2	0.186 ± 0.074	0.201 ± 0.032
Hcr group	1	0.996 ± 0.032	1.002 ± 0.044

### 2.4 Histopathological examination

Gill samples were dehydrated in increasing concentrations of ethanol (50%, 70%, 80%, 90% and 100%), placed in xylene for 1 minute, dried, and embedded in paraffin. Gill tissue samples were cut into thin slices of 2-6 μm thickness using a rotary slicer (Leica RM2235). Use haematoxylin and eosin and stain according to the manufacturer’s instructions. Observations were made using an Olympus BX53 research-grade biomicroscope (Olympus BX53, Japan), and gill sample changes were observed with cellSens software (cellSens 4.1) and photographed for preservation.

### 2.5 Histological enzyme activity

Assays of hydrogen peroxide (H_2_O_2_), superoxide dismutase (SOD), glutathione peroxidase (GSH-PX), total antioxidant capacity (T-AOC), catalase (CAT), and malondialdehyde (MDA) levels in the gills were performed according to the manufacturer’s instructions. The activities of Na^+^K^+^-ATPase, Ca^2+^-ATPase, and Mg^2+^-ATPase were determined with an ATPase Assay Kit (Kit No. A016-2-2). All kits were purchased from Nanjing Jiancheng Bioengineering Institute of China (Nanjing, China).

### 2.6 Gill metabolome analysis

The untargeted metabolomics assay was performed by LC-Bio (Hangzhou, China) with the following procedures: (a) homogenization of 18 fish gills in liquid nitrogen; (b) Metabolite enrichment was performed by methanol-buffer precipitation (50%, v/v). The extracted samples were subjected to random machine sequential testing, and QC samples were inserted before, during and after the samples as a repetitive assessment of the experimental technique. Detection of eluted metabolites in columns by high performance liquid chromatography (HPLC). The data were preprocessed using XCMS (XCMS-v4.7) software. Initial feature annotation via MetaX-driven database comparison (PlantCyc/KEGG/HMDB). Identification of metabolites in product secondary mass spectra using in-house libraries. MetaX software was used to perform univariate and multivariate analyses of the metabolomics data to identify metabolites that were enriched by differences between groups (dm). Differentially abundant features were identified when meeting all criteria: (a) Presence in ≥2 biological replicates or relative abundance ≤50%; (b) Significant inter-group divergence (BH-corrected q < 0.05) via Wilcoxon rank-sum test; (c) OPLS-DA variable importance projection (VIP) score ≥1.0. KEGG pathway enrichment profiling was conducted using a hypergeometric distribution model. Functionally annotated terms with corrected P < 0.05 were designated as significantly enriched clusters for differentially expressed proteins. Gene set enrichment analysis was performed using GSEA (v4.1.0) and MSigDB software programs to determine whether a group of genes were differentially enriched in a specific KEGG pathway.

### 2.7 Transcriptome analysis

Gill tissue samples from three biological replicates per group were subjected to transcriptome sequencing through Hangzhou Lianchuan Biotechnology Company, Ltd (Hangzhou, China). Total RNA was extracted using TRIzol reagent (TaKaRa, Dalian, China), with RNA integrity verified by Bioanalyzer 2100 Bioanalyzer (RIN > 8.0). First-strand cDNA synthesis was performed using 6-base random hexamer primers with mRNA as template. Second-strand cDNA was generated by adding reaction buffer, RNase H, dNTPs, and DNA polymerase. The products were purified with QIAquick PCR Purification Kit (Qiagen) and eluted in EB buffer. AMPure XP beads (Beckman Coulter) and USER enzyme (NEB) were employed for size selection and degradation of uracil-containing second-strand cDNA, ensuring strand-specificity of the final library. PCR amplification was conducted using Phusion High-Fidelity DNA Polymerase (Thermo Scientific) with indexed primers. Sequencing was performed by LC-Bio Co., Ltd (Hangzhou, China) on Illumina NovaSeq 6000 platform. Then sequence quality was verified using FastQC (http://www.bioinformatics.babraham.ac.uk/projects/fastqc/ , 0.11.9). Genes differential expression analysis was performed by DESeq2 software between two different groups (and by dgeR between two samples). Differentially expressed genes (DEGs) were screened based on DESeq2, with significance thresholds set at |log_2_(fold change)| ≥ 1 and corrected *p* value < 0.05. Volcano plots were generated to demonstrate the differential expression distributions by Gplot2, and a hierarchical clustering heatmap was visualized using the pheatmap to visualize hierarchically clustered heatmaps of DEGs. The genes with the parameter of false discovery rate (FDR) below 0.05 and absolute old change ≥ 2 were considered differentially expressed genes. The differentially expressed genes were then analyzed for GO function and KEGG pathway enrichment.

### 2.8 Gene ontology and enrichment exploration

GO enrichment analysis of DEGs was performed using Wallenius non-central hypergeometric distribution. Pathway enrichment analysis was performed using the KEGG database to further assess significantly enriched metabolic or signaling pathways, with *p* value < 0.05 being significantly enriched for DGE.

### 2.9 qRT-PCR validation

Total RNA was isolated from frozen gills using the TRIzol Reagent Kit according to the instructions of the manufacturer and assessed for quality. Samples with A260/A280 RNA ≥ 1.8 were selected for cDNA synthesis according to the manufacturer’s instructions (Beijing Tiangen). The primers used are listed in **Supplementary Table S1**. Referring to the previous study by Lu et al. (23), specific primers were designed using the online tool Primer 3 plus (https://www.primer3plus.com/) based on the Amur grayling transcriptome sequence (PRJNA907151). qRT-PCR analysis was performed using the SYBR Premix Ex Taq II kit (Tli RNaseH Plus, Takara Bio, Japan) following the manufacturer’s thermal cycling parameters. The *β* actin served as an internal control to normalize the data. 2^-ΔΔCT^ method was used to calculate the relative expression of target genes (24).

### 2.10 Statistical analysis

All data are expressed as mean ± standard deviation (SD). One-way statistical analysis of variance (ANOVA) was performed using SPSS 20.0 (SPSS, Chicago, IL, USA). All data were normally distributed and passed the equal variance test. Tukey’s multiple *post hoc* test, and for the same sampling intervals, different letters indicate that the differences are significant (*p* < 0.05). Graphical representation of the experimental data was performed using GraphPad Prism 9.0 (GraphPad Software, USA).

## 3 Results

### 3.1 Accumulation of Cr in tissues

**Figure 1:**
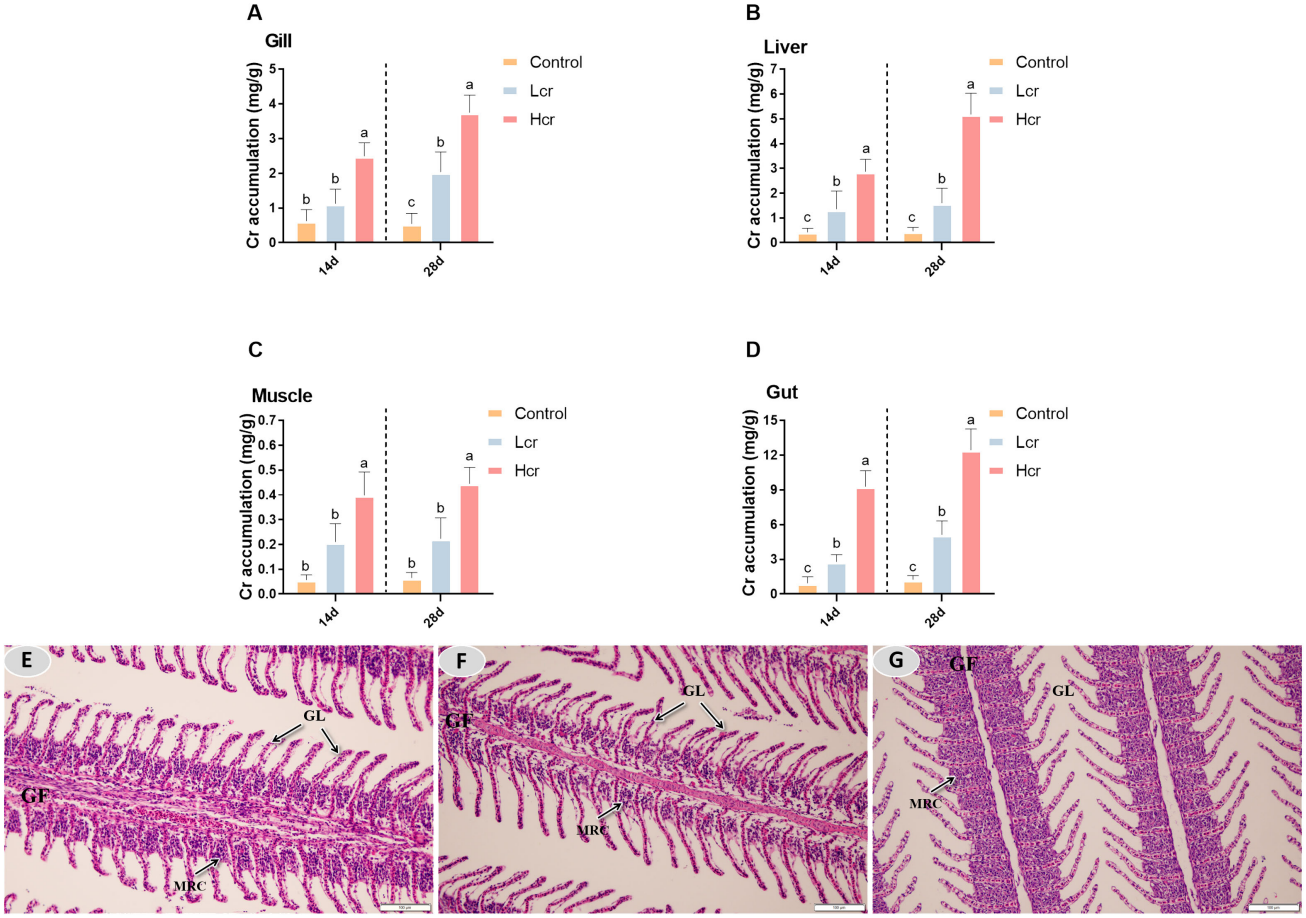
After 14 and 28 days, different levels of Cr^6+^ accumulated in gills, liver, muscle and intestine of the *T. grubii* **(A-D)**. The data are expressed as the means ± S.D (n = 6). Bars with different letters are significantly different (*P* < 0.05) according to Tukey’s test for the same sampling interval. Gill tissue changes in *T. grubii* after 28 days of Cr^6+^ exposure **(E-G)**. **(E)** H&E staining in the control group; scale bars = 100 µm. **(F)** H&E staining in the Lcr group; scale bars = 100 µm. **(G)** H&E staining in the Hcr group; scale bars = 50 µm 100 µm. GF (gill filaments), GL (gill lamellae), MRC (mitochondria-rich cell).

As shown in **Figure 1**, Cr^6+^ accumulated in the gills, liver and intestines of the fish at Days 14 and 28. The level of Cr^6+^ accumulation in fish increased in a concentration-dependent manner (*P* < 0.05). The accumulation level of Cr^6+^ in each tissue was in the order of intestine>liver>gill>muscle. Compared with those in the control group, the Cr^6+^ accumulation levels in the gill, liver, intestine and muscle in the Hcr group were significantly increased (*P* < 0.05), whereas Cr^6+^ accumulation in the muscle did not significantly differ between the Lcr and control groups (*P* > 0.05).

### 3.2 Histological analysis of gill tissues using H&E-stained sections

After 28 days, the gill filaments of the control group remained intact, the gill lamellae were neatly arranged, and complete physiological structures were observed (**Figure 1E**). Gill filament mitochondria-rich cells (MRCs) were partially vacuolated and gill filament thickness was significantly reduced in the Lcr group compared to the control group (**Figure 1F**). Compared with those of the control group, the Hcr group gill filaments of MRCs were heavily vacuolated, the gill filament thickness was significantly reduced, gill lamellae were significantly thinner, and epithelium vascular congestion was observed (**Figure 1G**).

### 3.3 Exposure to Cr induces changes in enzyme activity

**Figure 2:**
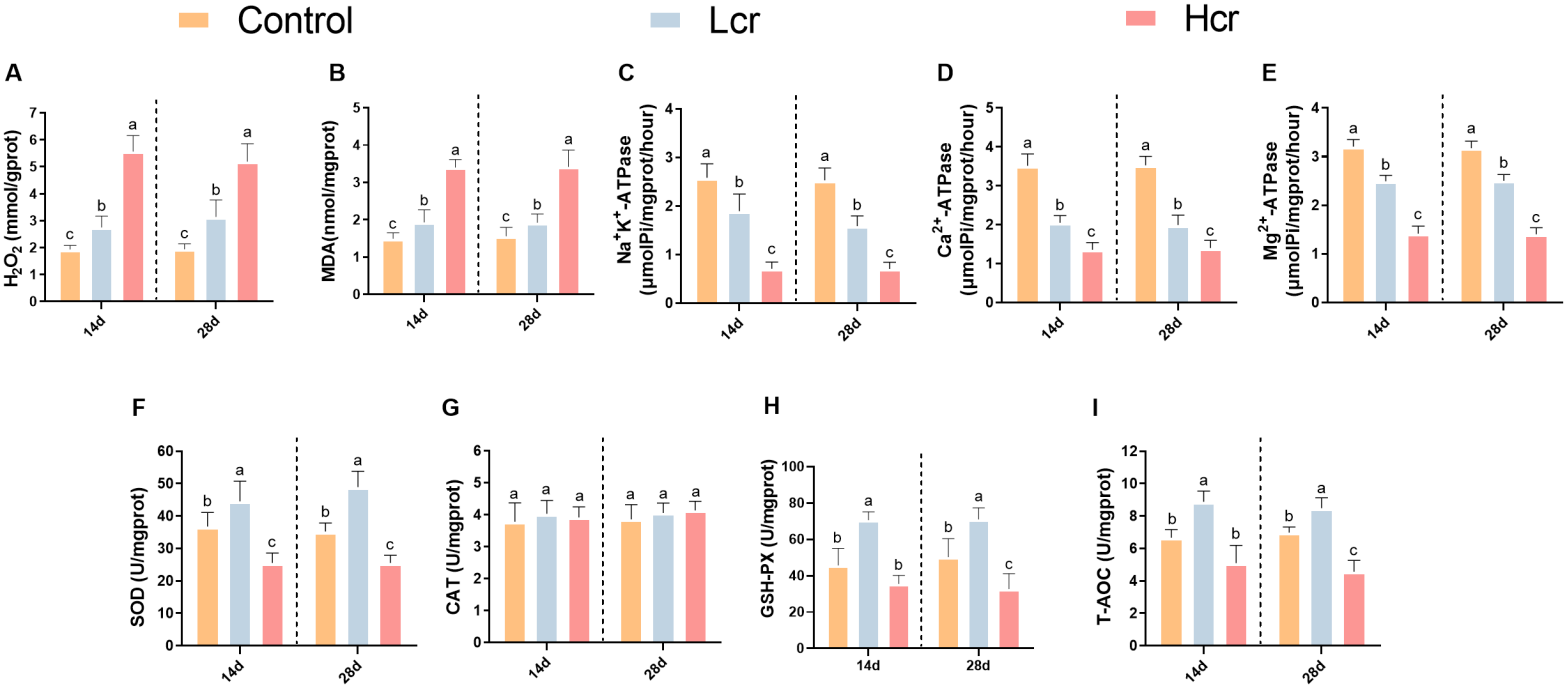
Changes in the gill enzyme activity indices of *T. grubii* after 14 and 28 days of treatment with different concentrations (0.2 and 1 mg/L) of Cr^6+^ stress. **(A)** H_2_O_2_; **(B)** MDA; **(C)** Na^+^K^+^-ATPase; **(D)** Ca^2+^-ATPase; **(E)** Mg^2+^-ATPase; **(F)** SOD; **(G)** CAT; **(H)** GSH-Px; **(I)**: T-AOC. The data are expressed as the means ± SDs (n=6). Bars with different letters are significantly different (*P* < 0.05) according to Tukey’s test for the same sampling interval.

To investigate whether exposure to Cr^6+^ causes gill damage, we examined the levels of MDA and H_2_O_2_ in gill tissue (**Figures 2A, B**). Compared with the control group, H_2_O_2_ levels were significantly greater in both the Lcr and Hcr groups (*P* < 0.05). Compared with those in the control group, the MDA levels were significantly greater in the Hcr group (*P* < 0.05). However, there was no significant difference in the MDA levels between the control and Lcr groups (*P* > 0.05).

We further measured changes in gill enzyme activity indicators using assay kits (**Figures 2C-E**). Compared with those in the control group, the Na^+^K^+^-ATPase, Ca^2+^-ATPase, and Mg^2+^-ATPase activities decreased with increasing Cr^6+^ levels (*P* < 0.05). Compared with those in the control group (**Figures 2F-I**), the SOD, GSH-PX, and T-AOC activities increased but then decreased with increasing Cr^6+^ concentration (*P* < 0.05). However, the CAT activity did not significantly differ between the groups (*P* > 0.05).

### 3.4 Metabolomics analysis of the effects of Cr^6+^ exposure on gill metabolism

**Figure 3:**
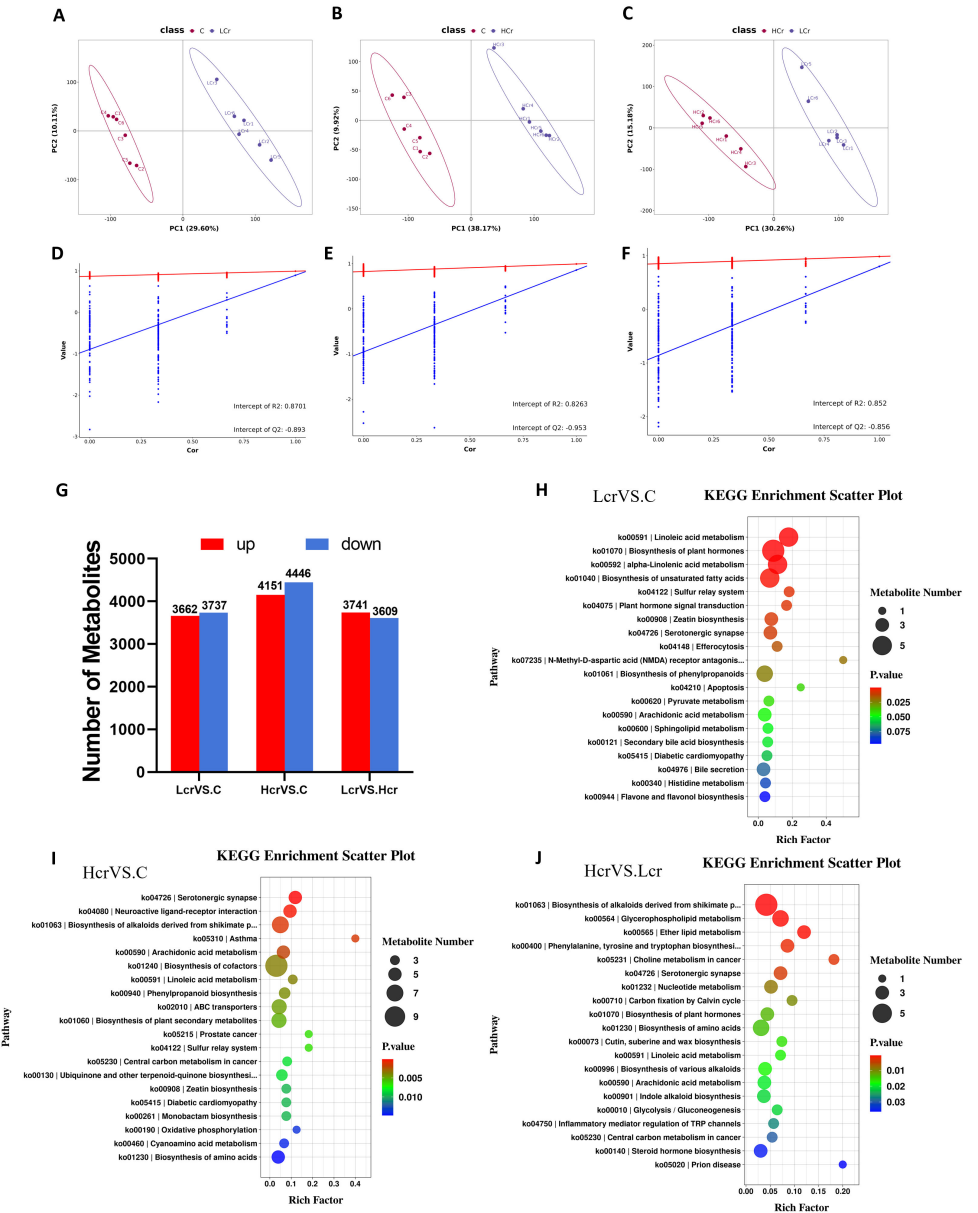
Quality analysis of metabolomics data and KEGG enrichment analysis (n=6). OPLS-DA score plots for metabolites in the C, Lcr and Hcr groups **(A–C)**. OPLS-DA permutation test for the positive and negative ion modes **(D)** C vs. Lcr; **(E)** C vs. Hcr; **(F)** Lcr vs. Hcr). Changes in the total amount of differential metabolites between the two groups **(G)** KEGG enrichment analysis between the two groups **(H)** C vs. Lcr; **(I)** C vs. Hcr; **(J)** Lcr vs. Hcr).

Metabolomics was used to further clarify the negative effects of chronic exposure to Cr^6+^ on *T. grubii* gill tissue. The OPLS-DA results revealed significant separation between each Cr^6+^ treatment group and the control group, suggesting that Cr^6+^ significantly interfered with the metabolic profile of gill tissues (**Figures 3A-C**). A permutation test chart was constructed to indicate the degree of model fit. The Q2 values were all <R2 values, and the intercepts of the Q2 values on the y-axis were all <0 (**Figures 3D-F**), indicating that the OPLS-DA model was not overfitted. Bar charts were constructed to display the number of differentially abundant metabolites (**Figure 3G**). **Supplementary Figure S1**. shows heat maps of the top 30 metabolites with the highest differential abundance in the Lcr and C groups. **Supplementary Figure S2**. shows heatmaps of the top 30 differentially abundant metabolites in the Hcr and C groups. **Supplementary Figure S3**. shows heat maps of the top 30 metabolites with the highest differential abundance in the Hcr, Lcr, and C groups.

Enrichment analyses of the KEGG pathway were performed on the differential metabolites (**Figures 3H-J**). Linoleic acid metabolism, biosynthesis of plant hormones, alpha-linolenic acid metabolism, biosynthesis of plant secondary metabolites, and the sulfur relay system were the five pathways most significantly enriched in the C Vs. Lcr group (**Figure 3H**). The serotonergic synapse, neuroactive ligand–receptor interaction, biosynthesis of alkaloids derived from the shikimate pathway, asthma, and arachidonic acid metabolism pathways were the five pathways most significantly enriched in the C Vs. Hcr group (**Figure 3I**). The biosynthesis of alkaloids derived from the shikimate pathway, glycerophospholipid metabolism, ether lipid metabolism, phenylalanine, tyrosine and tryptophan biosynthesis, and choline metabolism in cancer were the five pathways most significantly enriched in the Hcr Vs. Lcr group (**Figure 3J**).

### 3.5 Transcriptomic analysis of the effect of Cr^6+^ exposure on gene expression in fish gills

**Figure 4:**
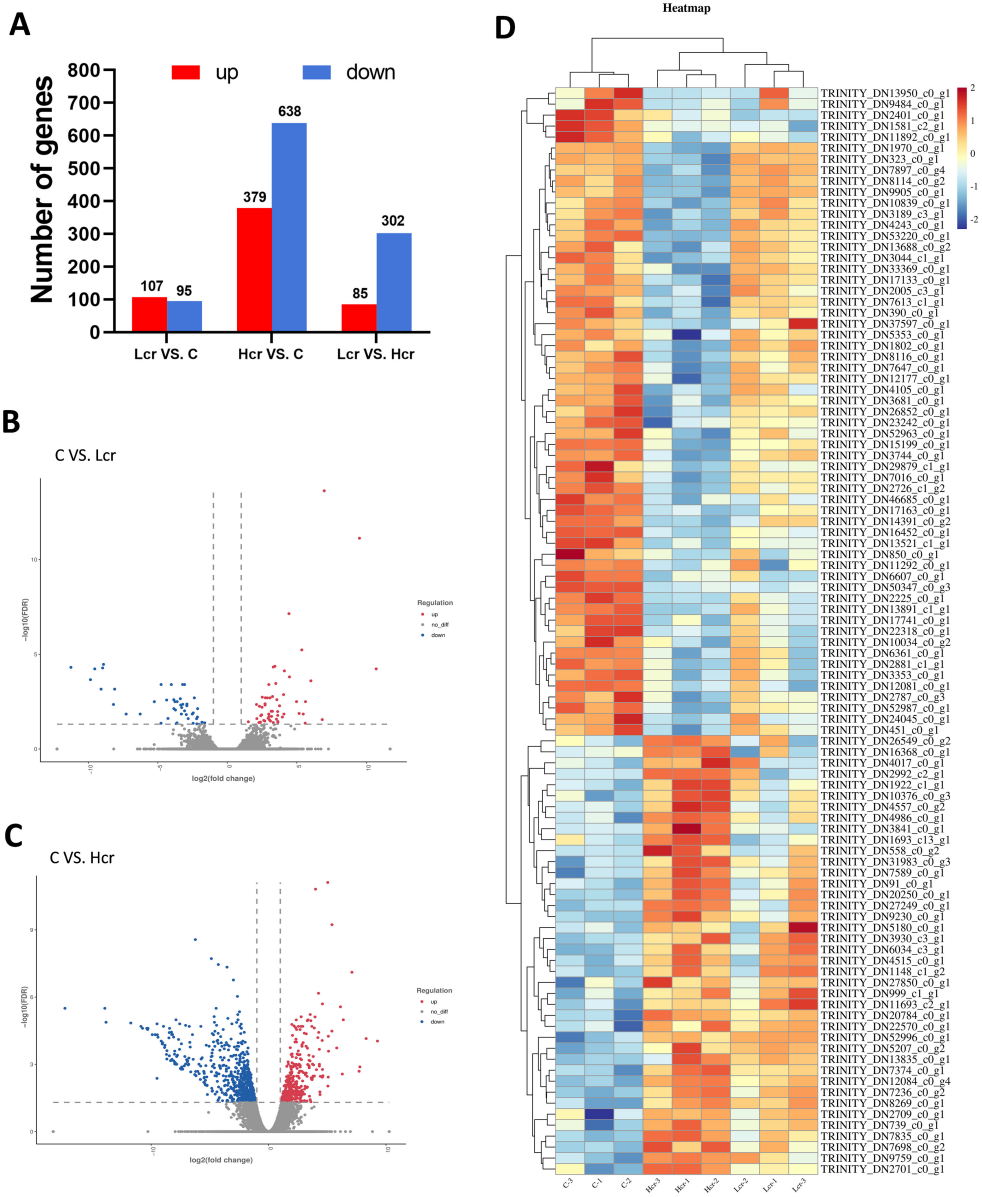
The results of mRNA sequencing. Differentially expressed genes **(A)**, volcano plots **(B, C)** and heatmaps **(D)** of the DEGs (P < 0.05). n = 3.

The levels of DEGs between the different treatment groups are shown in **Figure 4**, with 107 genes up-regulated and 95 genes down-regulated between the Lcr and C groups after Cr^6+^ exposure, and 379 genes were up-regulated and 638 genes were down-regulated between the Hcr and C groups. There were 85 genes upregulated and 302 genes downregulated between the Lcr and Hcr groups. **Figures 4B-D** show volcano plot and heatmap.

### 3.6 GO analysis and KEGG pathway enrichment based on DEGs

**Figure 5:**
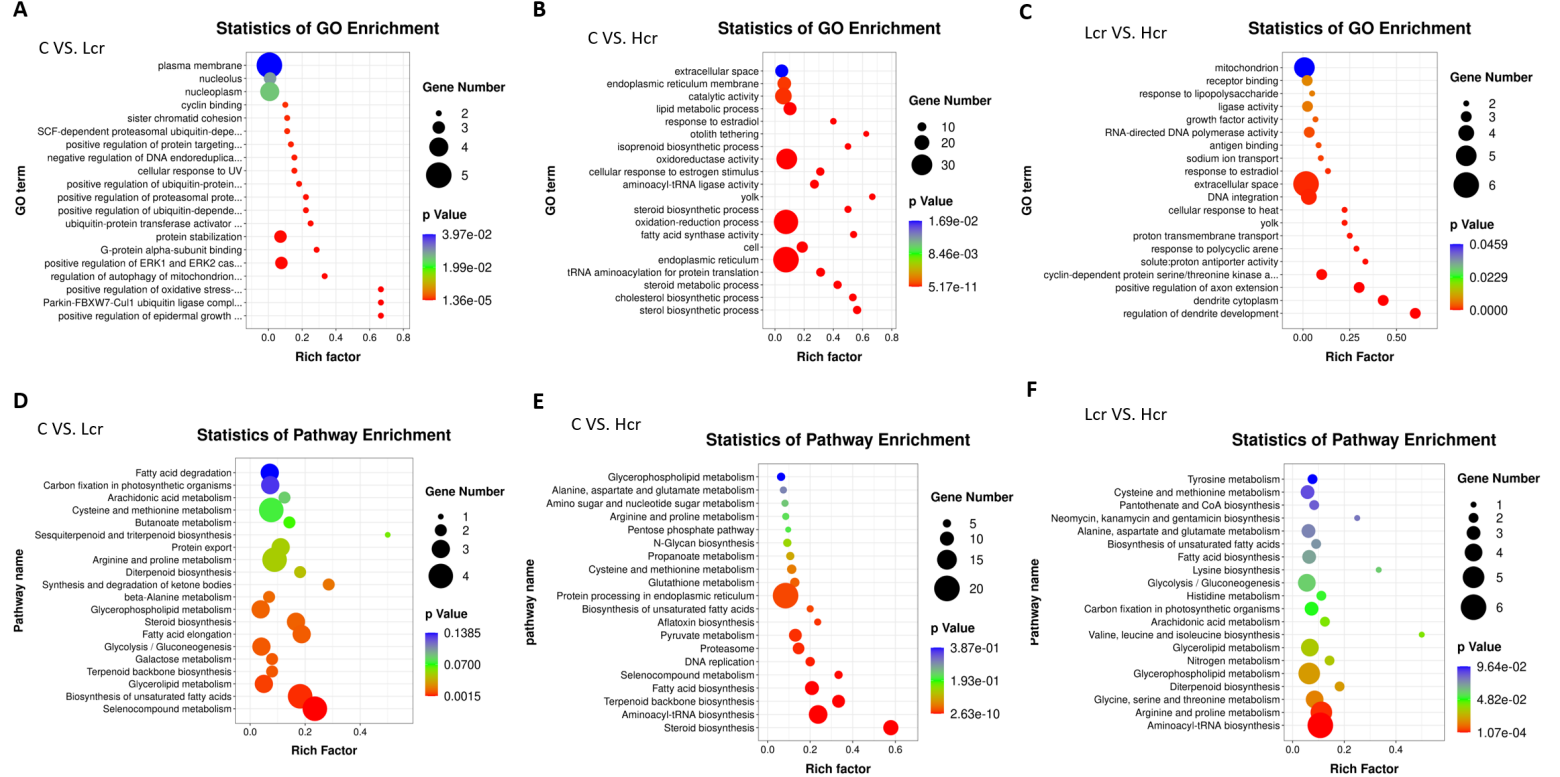
GO enrichment analysis of transcriptomic genes in gills. **(A)** C vs. Lcr; **(B)** C vs. Hcr; **(C)** Lcr vs. Hcr). KEGG enrichment analysis of transcriptomic genes in gills. **(D)** C vs. Lcr; **(E)** C vs. Hcr; **(F)** Lcr vs. Hcr).

The results obtained from GO analysis showed that positive regulation of oxidative stress-induced neuron intrinsic apoptotic signaling pathway, Parkin-FBXW7-Cul1 ubiquitin ligase complex, positive regulation of epidermal growth factor-activated receptor activity, regulation of autophagy of mitochondrion, and positive regulation of ERK1 and ERK2 cascade were the five most significantly enriched terms in the C and Lcr groups (**Figure 5A**). The sterol biosynthetic process, parkin-cholesterol biosynthetic process, steroid metabolic process, tRNA aminoacylation for protein translation, and endoplasmic reticulum were the five most significantly enriched terms in the C and Hcr groups (**Figure 5B**). The regulation of dendrite development, dendrite cytoplasm, positive regulation of axon extension, cyclin-dependent protein serine/threonine kinase activity, and solute: proton antiporter activity were the five most significantly enriched terms in the Lcr and Hcr groups (**Figure 5C**). **Supplementary Figure S4**. shows the clusters to which each enriched terms in the comparison group belongs.

This study used the KEGG database to annotate the enrichment pathway of DEGs (**Figures 5D-F**). The galactose metabolism, steroid biosynthesis, terpenoid backbone biosynthesis, selenocompound metabolism, and biosynthesis of unsaturated fatty acids were the five most significantly enriched signal pathway in the C and Lcr groups. The steroid biosynthesis, aminoacyl-tRNA biosynthesis, fatty acid biosynthesis, proteasome, and pyruvate metabolism were the five most significantly enriched signal pathway in the C and Hcr groups. In addition, signaling pathways such as selenocompound metabolism, aflatoxin biosynthesis, glutathione metabolism, and protein processing in endoplasmic reticulum were similarly significantly enriched. The aminoacyl-tRNA biosynthesis, arginine and proline metabolism, glycine, serine and threonine metabolism, diterpenoid biosynthesis, and glycerophospholipid metabolism were the five most significantly enriched signal pathway in the Lcr and Hcr groups.

### 3.7 Cr^6+^ induces changes in the expression of key genes in gills

**Figure 6:**
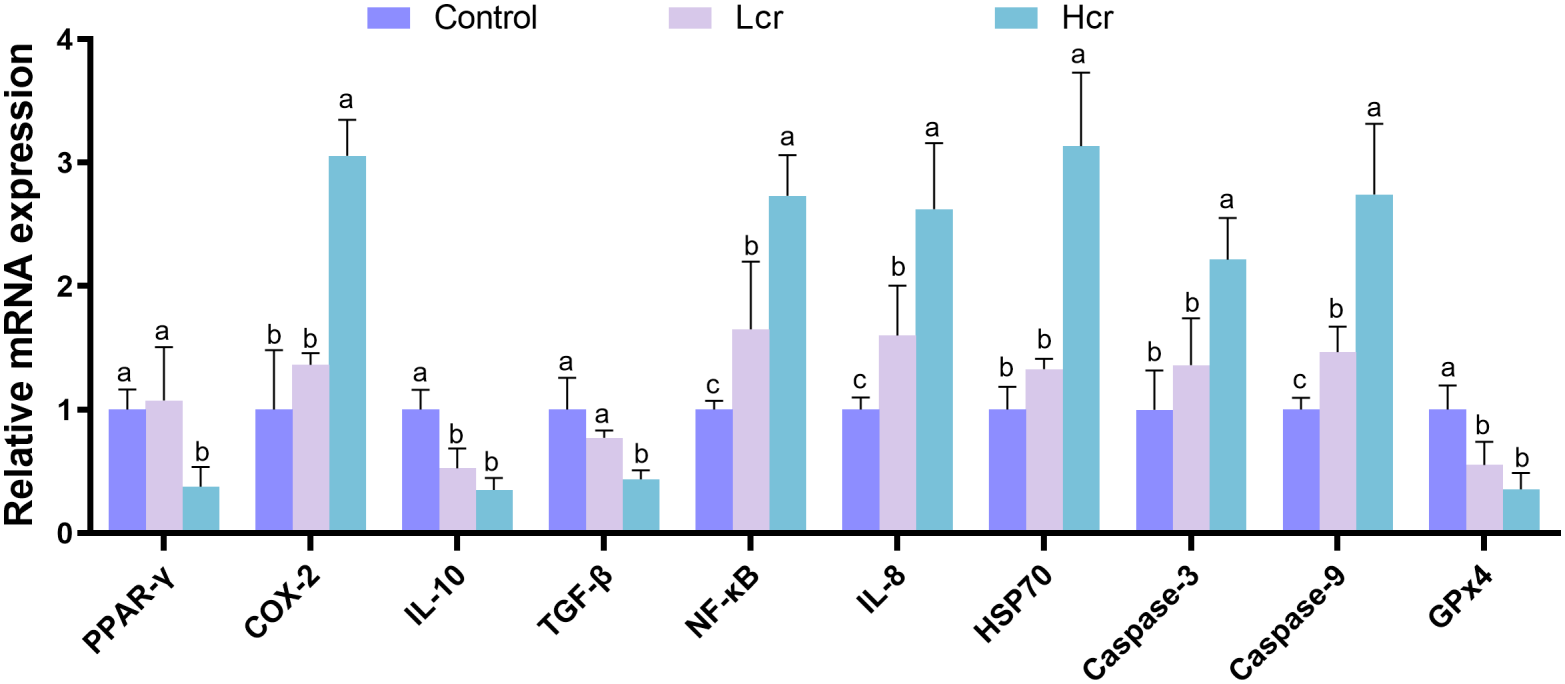
Cr^6+^ induces changes in the expression of key genes in gills. The data are expressed as the means ± S.Ds. (n = 3). The levels of peroxisome proliferator activated receptor (*PPAR)-γ*, Cyclooxygenase-2 (*COX-2*), interleukin 10 (*IL-10*), transforming growth factor-β (*TGF-β*), Nuclear factor κB (*NF-κB*), interleukin 8 (*IL-8*), 70-kDa heat shock protein (*Hsp70*), cysteinyl aspartate specific proteinase 3 (*Caspase-3*), cysteinyl aspartate specific proteinase 9 (*Caspase-9*), and Glutathione peroxidase 4 (*GPx4*) were detected. Bars with different letters are significantly different (*P* < 0.05) according to Tukey’s test for the same sampling interval.

To further explore the effects of Cr^6+^-induced oxidative stress on the expression regulation of genes in gill tissues, we validated the key genes by qRT–PCR (**Figure 6**). Compared with those in the control group, the expression levels of *IL-10* and *GPx4* were significantly lower, and those of *NF-κB*, *IL-8*, and *Caspase-9* were significantly greater in the Lcr group (*P* < 0.05). Compared with those in the control group, the expression levels of *PPAR-γ*, *IL-10*, *TGF-β* and *GPx4* were significantly lower, and those of *COX-2*, *NF-κB*, *IL-8*, *HSP70, Caspase-3*, and *Caspase-9* were significantly greater in the Hcr group (*P* < 0.05).

**Figure 7:**
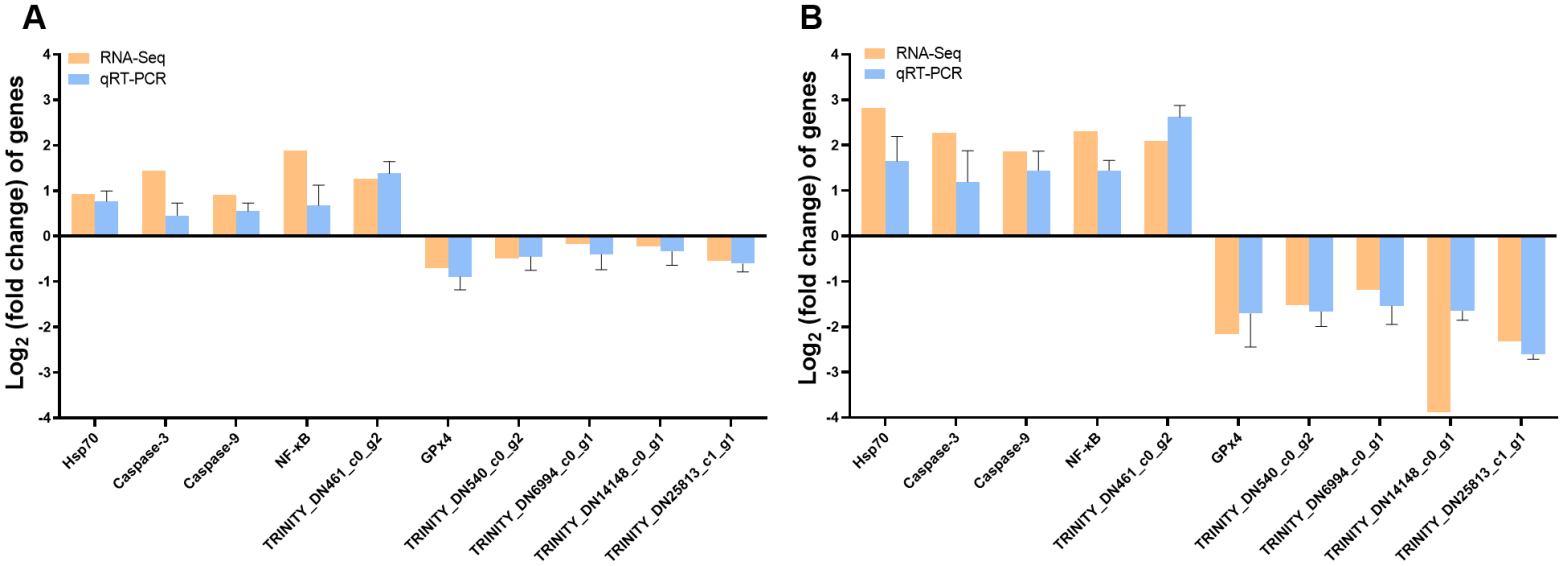
RT–PCR was used to validate transcriptomic gene expression. Comparison of genes for RNA-Seq and qRT–PCR **(A)** C vs. Lcr; **(B)** C vs. Hcr).

As shown in **Figure 7**, we selected 10 genes for gill qRT-PCR to verified the reliability and reproducibility of RNA-Seq. The results showed that the upregulation and downregulation trends of the genes in the qRT-PCR results were consistent with the RNA-Seq results, suggesting that the transcriptome sequencing data were reliable.

**Figure 8:**
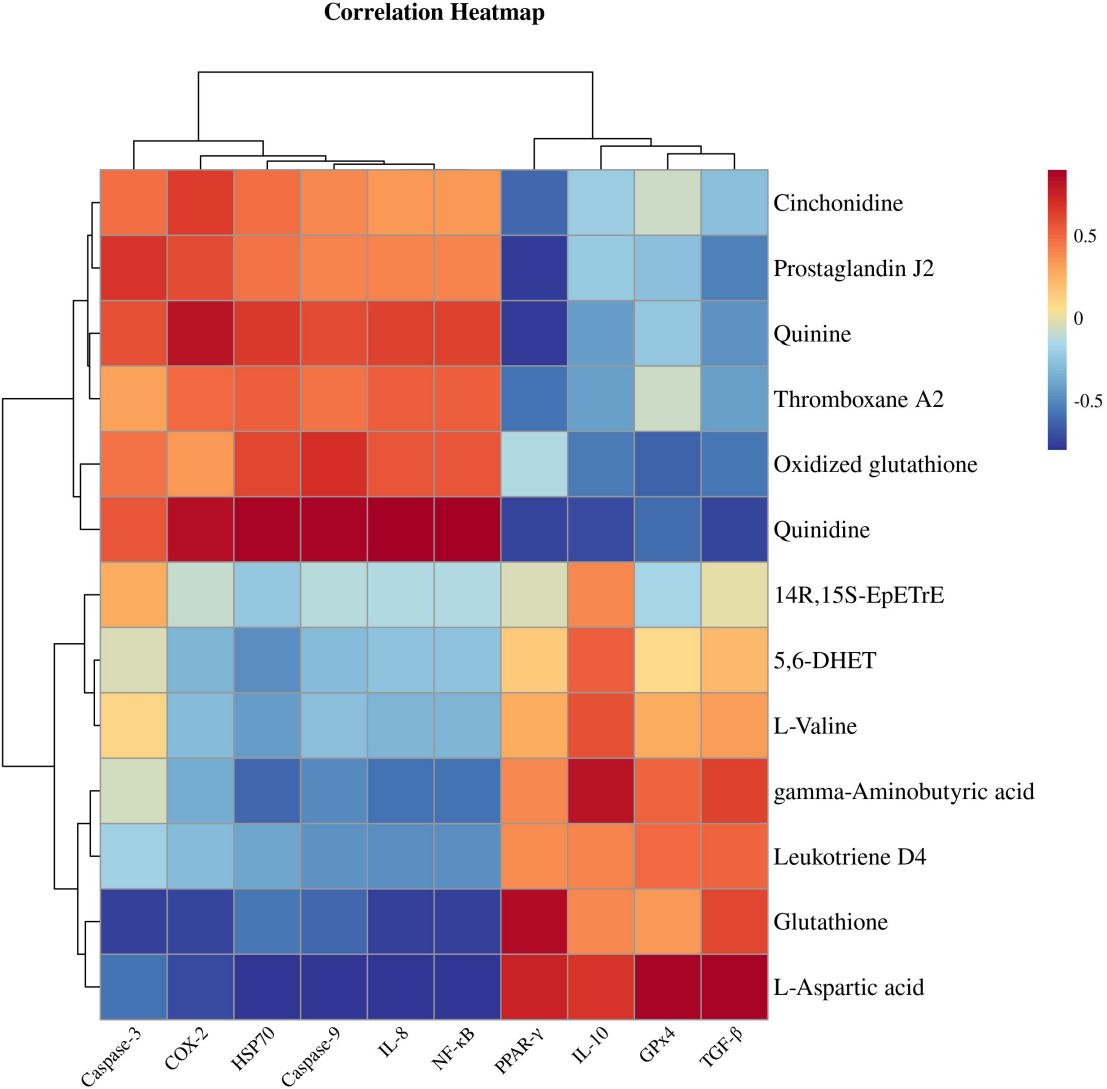
Heatmap for gene and metabolite correlation analysis.

**Figure 8** shows a heatmap for gene and metabolite correlation analysis. Correlation analysis in a clustered heatmap revealed that *PPAR-γ, IL-10, TGF-β* and *GPx4* were negatively correlated with the key metabolites cinchonidine, quinidine, quinine, 5-hydroxy-L-tryptophan, thromboxane A2 and prostaglandin J2 and positively correlated with 5,6-DHET, 14R,15S-EpETrE, γ-aminobutyric acid, leukotriene D4, L-aspartic acid, GABA, L- valine, L-tyrosine, and vanillin, whereas *COX-2, NF-κB, IL-8, HSP70, Caspase-3*, and *Caspase-9* were inversely correlated with the above metabolites.

## 4 Discussion

Aquatic ecosystems are at risk of multiple heavy metal exposures due to increased industrial wastewater discharges and agricultural pollution, of which Cr^6+^ is a representative persistent pollutant with significant toxic effects on aquatic animals through water column enrichment (1). As a key organ for respiration and ion exchange in fish, the gill is a primary target organ for heavy metal bioaccumulation (25). Therefore, a systematic analysis of the metabolic network remodeling of gill tissues under heavy metal exposure is highly valuable for revealing species-specific detoxification mechanisms and constructing ecological risk threshold models. Hexavalent chromium (CrVI), as a redox-active transition metal, induces ROS-mediated oxidative stress cascades through Fenton-like reactions, specifically depleting reduced glutathione (GSH) while elevating oxidized glutathione (GSSG) ratios (26). In this study, Cr^6+^ accumulated significantly in all tissues and organs with increasing exposure time. In the present study, Cr^6+^ accumulated significantly in all tissues and organs with increasing exposure time. Gills were in direct contact with water and were the primary target organ for Cr^6+^ enrichment. Intestine and liver had high metabolic activities so the Cr^6+^ accumulation was higher (19, 27). The main reasons for the significantly higher accumulation of Cr^6+^ in the intestine than in other organs may be twofold: firstly, the relatively limited absorption efficiency of Cr^6+^ in the intestine leads to the prolonged retention of Cr^6+^ in the intestinal lumen; secondly, cadmium is able to be transported and redistributed through the trans-organ interactions network (liver-intestinal axis, muscle-intestinal axis, brain-intestinal axis, etc.), which has the intestinal as its core, and ultimately, it forms a high-concentration accumulation in the intestinal tract (17, 28, 29). Two levels of Cr^6+^ caused damage to the gills of the fish, with the Hcr group being the most severely damaged. We found that Cr^6+^ can cause gill tissue damage mainly through vacuolization of MRC cells, congestion of epithelial vessels and a significant reduction in gill filament thickness. Previous studies have shown that Cr^6+^ exposure can lead to oxidative stress in fish through the liver-intestinal axis, resulting in liver damage and gut flora disruption (17). MDA and H_2_O_2_ are key indicators used to measure whether tissues are suffering from oxidative damage (30). The present experimental data revealed that Cr^6+^ exposure induced gill damage, and the concentrations of MDA and H_2_O_2_ in gill tissues were significantly and positively correlated with the dose of Cr^6+^ exposure. Although Cr^6+^ exposure led to a significant increase in H_2_O_2_ levels, CAT activity did not change significantly, probably because the organism had entered an irreversible stage of damage due to chronic Cr^6+^ stress, which is similar to the results of previous studies (20, 31). SOD, GSH-Px, and T-AOC levels tended to increase but then decreased with increasing Cr^6+^ content, possibly because *T. grubii* can adapt to low concentrations of Cr^6+^ by regulating its metabolism. However, when the Cr^6+^ concentration exceeded the tolerance threshold, the fish could not adapt to Cr^6+^ stress through their own metabolism, and the antioxidant defense system was imbalanced, which was similar to the findings of our previous study (17).

Owing to industrial pollution and emissions from agricultural activities, Cr^6+^ enters water bodies through soil infiltration, seriously threatening the survival of aquatic organisms. Cr^6+^ can accumulate in aquatic animals to produce toxic effects. In this study, we used a nontargeted metabolomics system to analyze the effects of Cr^6+^ on the metabolic regulatory network of gill tissues in *T. grubii*, aiming to elucidate the molecular toxicological mechanisms involved in the induction of oxidative stress, imbalance of lipid metabolism, and cellular inflammatory responses and to provide a theoretical basis for the risk assessment of Cr^6+^ contamination in aquatic ecosystems. In our study, Cr^6+^ exposure significantly elevated the metabolites thromboxane A2 and prostaglandin J2. The arachidonic acid metabolic pathway, serotonergic synapses, and neuroactive ligand–receptor interactions are central hubs for the regulation of inflammation, neurotransmitter systems, and cellular homeostasis. Studies have reported that heavy metal exposure can produce an inflammatory response through the metabolism of arachidonic acid to produce excess thromboxane A2 (32, 33). Maria is similar to our results in that activated microglia produce large amounts of PGs after neuronal injury. whereas Prostaglandin J2, the most toxic component of the PGs family, may contribute to many neurodegenerative disorders, *PPARγ* activation is associated with anti-inflammatory and neuroprotective signaling (34). In addition, cadmium exposure can also lead to significant downregulation of 5,6-DHET and 14R,15S-EpETrE metabolites in rats (35), which is similar to our findings that Cr^6+^ exposure leads to significant downregulation of these two metabolites. Heavy metals interfere with serotonergic synapses and GAD, reduce γ-aminobutyric acid (GABA) synthesis, lead to neuroexcitotoxicity, and can interact with arachidonic acid metabolism (36, 37). Leukotriene D4 alleviates inflammatory responses by activating the phospholipase C/Ca^2+^/protein kinase C pathway (38). L-Aspartic acid is another important amino acid that serves as an intermediate in the tricarboxylic acid (TCA) and urea cycles, attenuating external oxidative stress-induced tissue damage and inflammatory responses (39). Gamma-aminobutyric acid (GABA) is also an important amino acid with antioxidant properties that regulates lipid metabolism (40). In our study, chronic Cr^6+^ exposure resulted in significant increases in the levels of 5-hydroxy-L-tryptophan, thromboxane A2, and prostaglandin J2 and significant decreases in the levels of 5,6-DHET, 14R,15S-EpETrE, GABA, leukotriene D4, L-aspartic acid, and GABA. This suggests that Cr^6+^ exposure leads to arachidonic acid metabolism disruption and toxic action, whereas elevated levels of 5,6-DHET may be adaptive responses produced by the organism. In our study, the biosynthesis of alkaloids derived from the shikimate pathway was significantly altered, and 5-hydroxy-L-tryptophan, cinchonidine, quinidine, and quinine were significantly elevated, whereas L-alanine, L-tyrosine, and vanillin levels were significantly reduced. Cinchonidine protects against cisplatin-induced oxidative stress by activating the PI3K/AKT pathway (41). In contrast, some drugs, such as quinine and quinidine, have indirect antioxidant effects, thereby mitigating oxidative damage (42, 43). L-Valine promotes fish growth, improves antioxidant capacity and alleviates endoplasmic reticulum stress (44). Thus, the present study demonstrated that chronic Cr^6+^ exposure leads to disturbances in arachidonic acid metabolism and amino acid metabolism, induces oxidative damage to gill tissues, and regulates redox levels through metabolic reprogramming.

Fish gill tissue is an important osmoregulatory organ (45). When gill tissue is damaged, its osmoregulatory capacity is disrupted, directly affecting ATPase activity and leading to electrolyte imbalance (46). In the present study, we found that Cr^6+^ exposure resulted that steroid biosynthesis, fatty acid biosynthesis, biosynthesis of unsaturated fatty acids, aflatoxin biosynthesis, and lutathione metabolism signaling pathways were significantly altered. In addition, elevated expression of *HSP70*, which functions as oxidative stress biomarker, indicated that the cells were in a state of oxidative damage. Studies have shown that heavy metals and physiological stress can induce significant expression of stress proteins such as *HSP70* and promote apoptosis by catalyzing *Caspase-3* and *caspase-9* (47, 48). The experimental data revealed that chronic Cr^6+^ exposure significantly upregulated the expression of *HSP70*, activated the apoptotic factors *Caspase-3* and *Caspase-9* in gill tissues, and inhibited the expression of the key antioxidant regulator *GPx4*, suggesting that Cr^6+^ exposure mediates structural damage in gill tissues. The peroxisome proliferator-activated receptor (PPAR) family, classified within the ligand-activated nuclear receptor superfamily, comprises three functionally distinct isoforms designated as α, β/δ, and γ subtypes (49).These nuclear receptors play important roles in metabolism, cell differentiation and inflammatory processes. *PPARγ* has been shown to be a key factor in the regulation of lipid metabolism and adipocyte differentiation, and increasing evidence suggests that *PPARγ* exerts anti-inflammatory and neuroprotective functions by regulating the transcription of inflammatory genes (50, 51). In addition, previous studies have found that Cr^6+^ can cause severe oxidative damage to tissues and organs throughout the body via the blood circulation (52). Alwaili et al. found that Cr^6+^ exposure inhibits *PPARγ* expression levels and mediates inflammatory responses in the mouse heart (53). Jin et al. found that Cr^6+^ exposure inhibited *PPARγ* expression levels and mediated apoptosis in chicken pancreas (54). Notably, Cr^6+^ exposure can lead to blood-brain barrier damage and brain-hepatic axis neurotoxicity in zebrafish and snakehead fish (28). The results of the present study showed that chronic Cr^6+^ exposure resulted in significant inhibition of *PPARγ*, accompanied by decreases in the expression levels of its downstream anti-inflammatory mediators *TGF-β* and *IL-10*. Moreover, Cr^6+^ promotes the expression of *COX-2* and induces the release of the proinflammatory cytokine *IL-8* via the NF-κB pathway, which ultimately triggers a cascading inflammatory response. Low *PPAR-γ* expression deregulates *COX-2* inhibition and activates the NF-κB signaling pathway to promote inflammatory progression (55). *COX-2* synthesizes and promotes the formation of thromboxane A2 and prostaglandin E2, leading to disorders of arachidonic acid metabolism and causing inflammation and nerve damage (56, 57). Cinchonine inhibits *PPARγ* expression, downregulates fatty acid synthesis, and modulates inflammatory responses (58). *IL-10* and *TGF-β1* achieve anti-inflammatory effects through transcriptional silencing of pro-inflammatory cytokines and constitutive inhibition of NF-κB nuclear translocation, thereby attenuating inflammation-driven tissue damage (59, 60). *IL-8* can be activated by the NF-κB pathway to promote inflammation (61). These findings are similar to those in our study, where correlation analyses revealed that *PPAR-γ* was negatively correlated with cinchonidine, quinidine, quinidine, 5-hydroxy-1-tryptophan, thromboxane A2, and prostaglandin J2, whereas the anti-inflammatory factors *IL-10, TGF-β*, and *GPx4* were downregulated, and proinflammatory factors were upregulated. However, *COX-2* showed the opposite trends of correlation with the above metabolites. GABA exerts anxiolytic effects as a neurotransmitter while inhibiting the release of proinflammatory factors and regulating lipid metabolism by enhancing *PPAR-γ* (6, 62). In the present study, we analyzed the adverse effects of Cr^6+^ exposure on gill tissues by integrating transcriptomics and metabolomics. We found that Cr^6+^ exposure significantly led to increased glutathione depletion, resulting in decreased *GPX4* expression levels. In addition, Cr^6+^ exposure significantly inhibited the expression level of *PPAR-γ*, a key factor in lipid metabolism, and induced lipid metabolism disorders, which further activated the NF-κB pro-inflammatory signaling pathway to aggravate the release of inflammatory factors and triggered the Caspase-3 cascade reaction to trigger apoptosis. In this study, we confirmed the mechanism of action of Cr^6+^ exposure in inducing gill toxicity in *Thymallus grubii*. Cr^6+^ accumulated in tissues such as intestine, liver and gill, and these results highlight the multi organ distribution pattern of Cr^6+^. Based on these results, we will systematically investigate the cross-organ interaction network of Cr^6+^ to elucidate the mechanism of Cr^6+^ induced oxidative stress cascade in aquatic animals.

## 5 Conclusion

**Figure 9:**
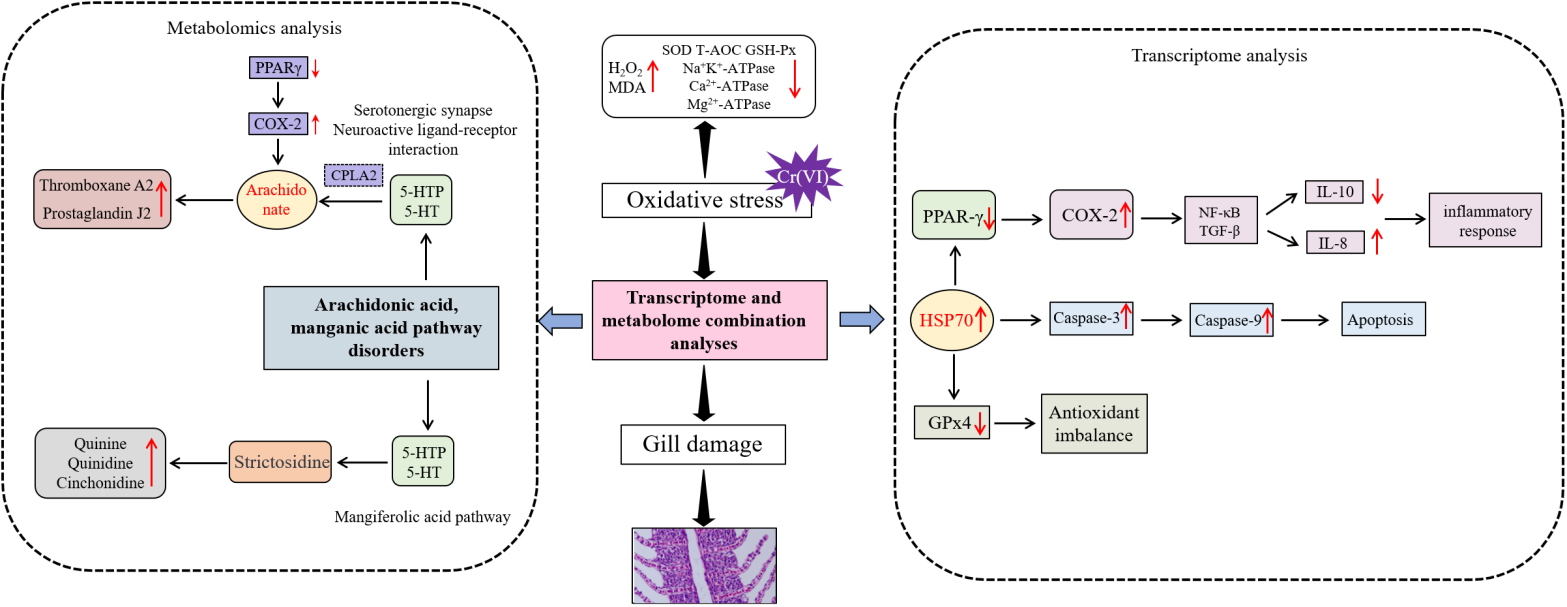
Mechanism of Cr^6+^ causing gill metabolic disorders.

In conclusion, this study elucidates for the first time the mechanism of gill tissue damage caused by Cr^6+^ in aquatic organisms. The data suggest that Cr^6+^ exposure disrupts the arachidonic acid metabolic pathway by inactivating *PPAR-γ* to deregulate *COX-2*, leading to oxidative stress and inflammatory responses. Disturbances in arachidonic acid metabolism led to the release of proinflammatory mediators such as thromboxane A2 and prostaglandin J2, which are accompanied by the accumulation of oxidized glutathione. *PPAR-γ* activity inhibition leads to blocked synthesis of metabolites with anti-inflammatory/antioxidant functions, such as GABA, quinidine, and L-aspartic acid. Therefore, targeted activation of *PPAR-γ* combined with *COX-2* inhibitors could be a potential intervention strategy to reverse Cr^6+^ toxicity (**Figure 9**).

## Data Availability

The datasets presented in this study can be found in online repositories. The names of the repository/repositories and accession number(s) can be found in the article/**Supplementary Material**.
