# Correction: Chronic hexavalent chromium exposure induces oxidative stress-mediated molecular cascades in Thymallus grubii gills: evidence from integrated transcriptomics and metabolomics

**DOI:** 10.3389/fimmu.2025.1692659

**Published:** 2025-09-04

**Authors:** Xinchi Shang, Xinghua Che, Kai Ma, Bo Ma, Huizhi Sun, Wenhua Wu, Hailong He, Meiqi Xing, Wei Xu, Yongquan Zhang

**Affiliations:** ^1^ Key Open Laboratory of Cold Water Fish Germplasm Resources and Breeding of Heilongjiang Province, Heilongjiang River Fisheries Research Institute, Chinese Academy of Fishery Sciences, Harbin, China; ^2^ College of Life Science, Northeast Agricultural University, Harbin, China; ^3^ College of Marine Biology and Fisheries, Hainan University, Haikou, China; ^4^ Heilongjiang Aquatic Animal Resource Conservation Center, Harbin, Heilongjiang, China

**Keywords:** gill, metabolome, transcriptome, Cr(VI) stress, inflammatory responses

Affiliation “Key Open Laboratory of Cold Water Fish Germplasm Resources and Breeding of Heilongjiang Province, Heilongjiang River Fisheries Research Institute, Chinese Academy of Fishery Sciences, Harbin, China” was missing institute details and was displayed as “Heilongjiang River Fisheries Research Institute, Chinese Academy of Fishery Sciences, Harbin, China”.

The original version of this article has been updated.

