# Supplementary figures and images for: Chronic hexavalent chromium exposure induces oxidative stress-mediated molecular cascades in Thymallus grubii gills: evidence from integrated transcriptomics and metabolomics
Source: Front Immunol. 2025 Jul 9;16:1633174. doi: 10.3389/fimmu.2025.1633174 (PMC12285536; doi:10.3389/fimmu.2025.1633174)

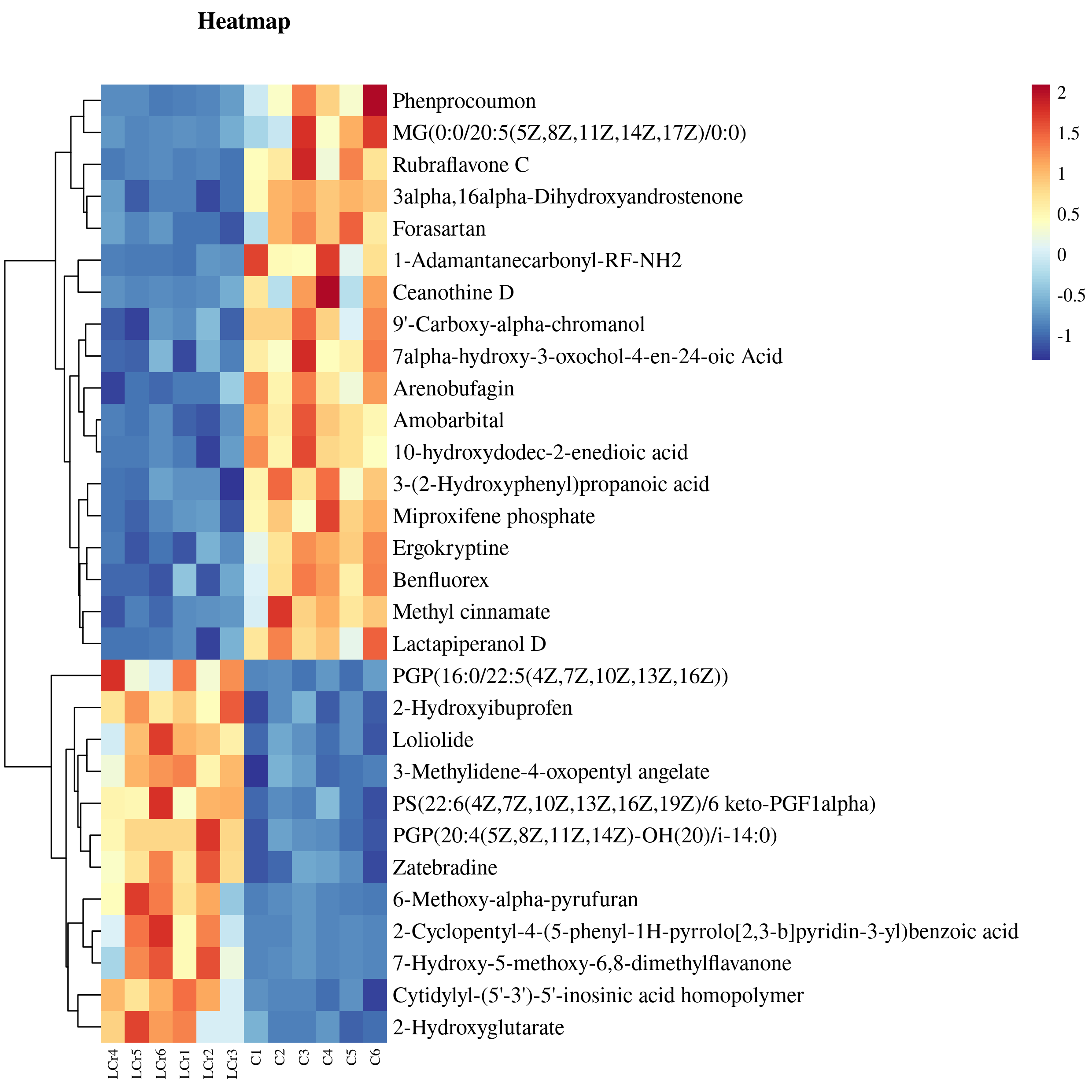

Supplement: Supplementary file 1 [file Image1.tiff]

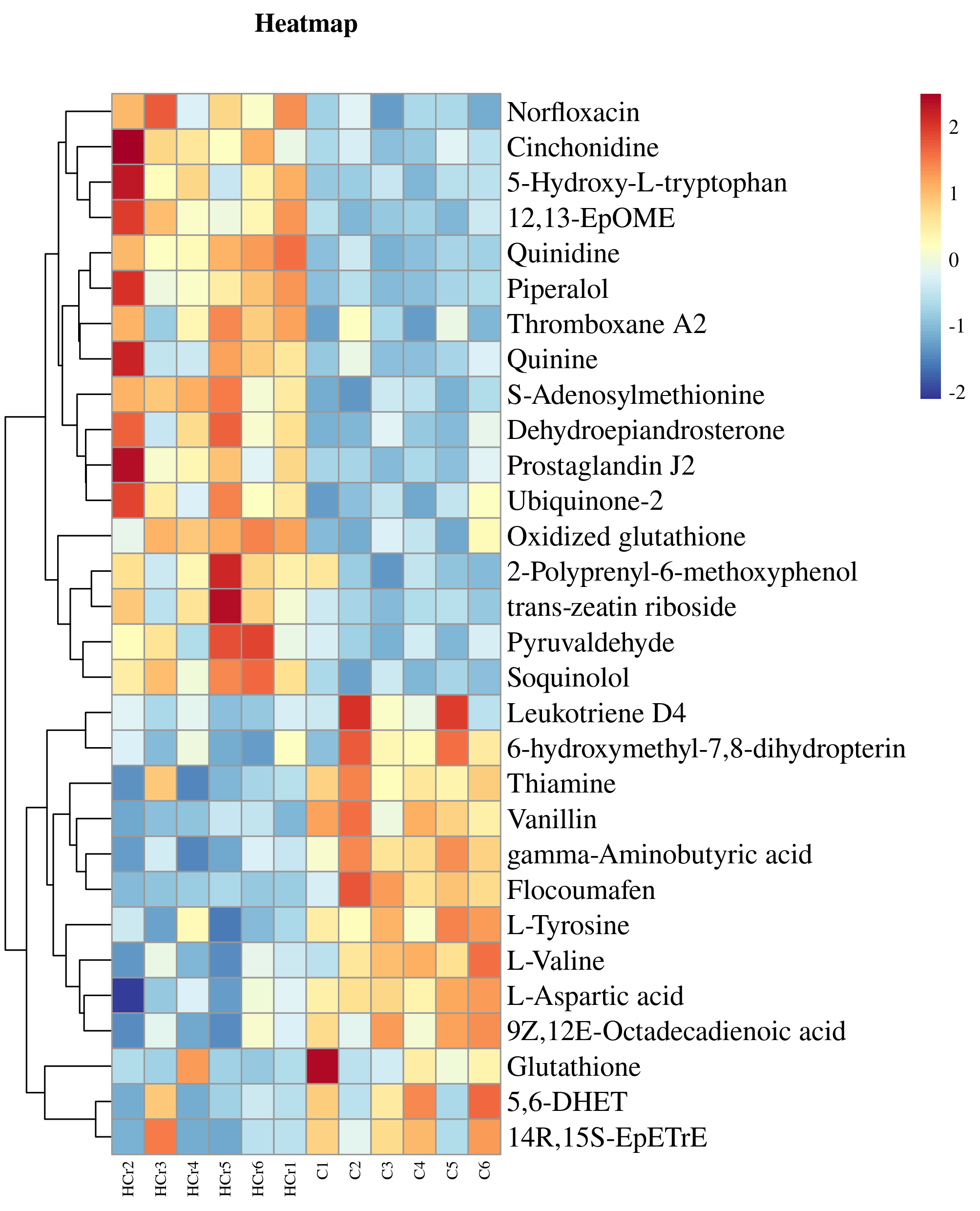

Supplement: Supplementary file 2 [file Image2.tiff]

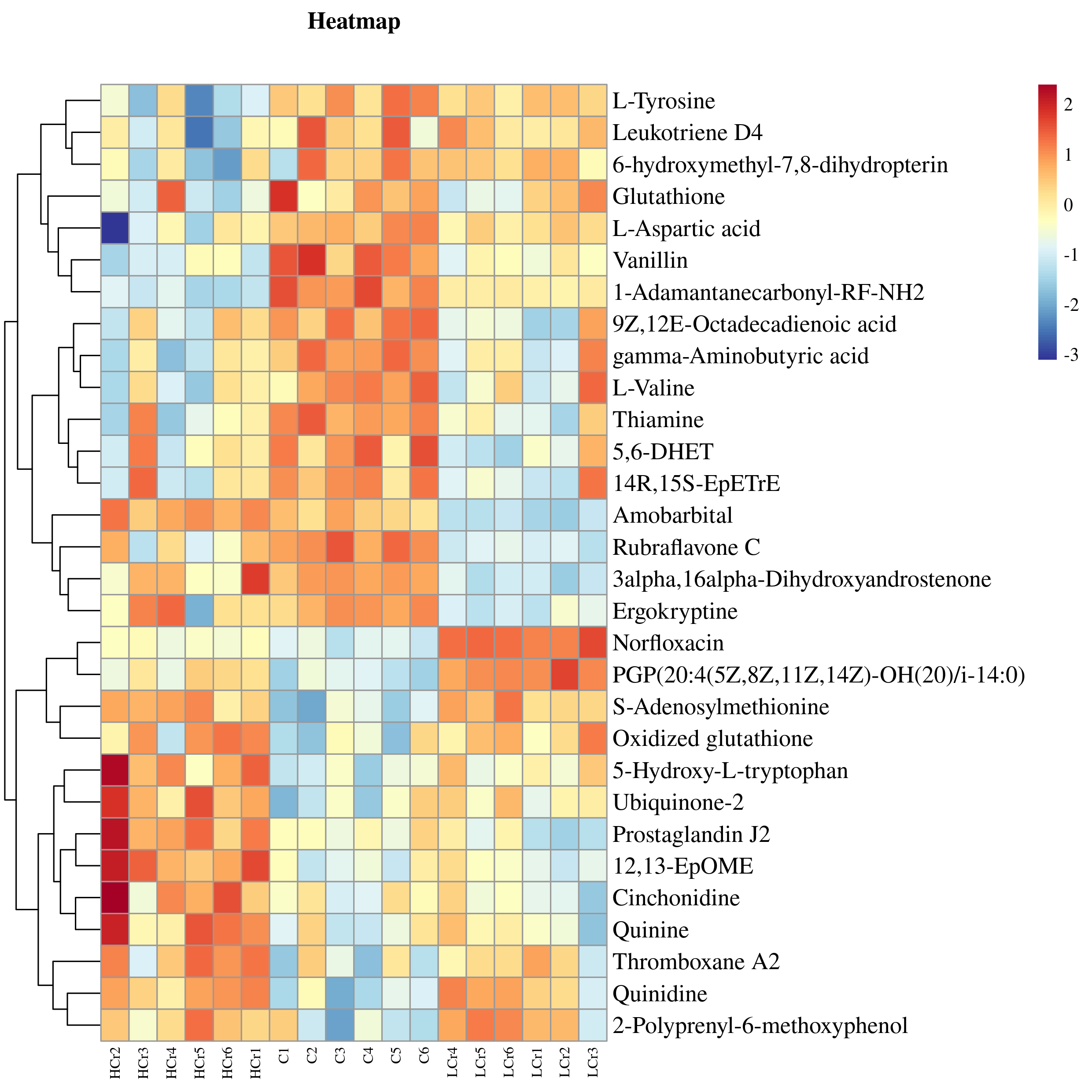

Supplement: Supplementary file 3 [file Image3.tiff]

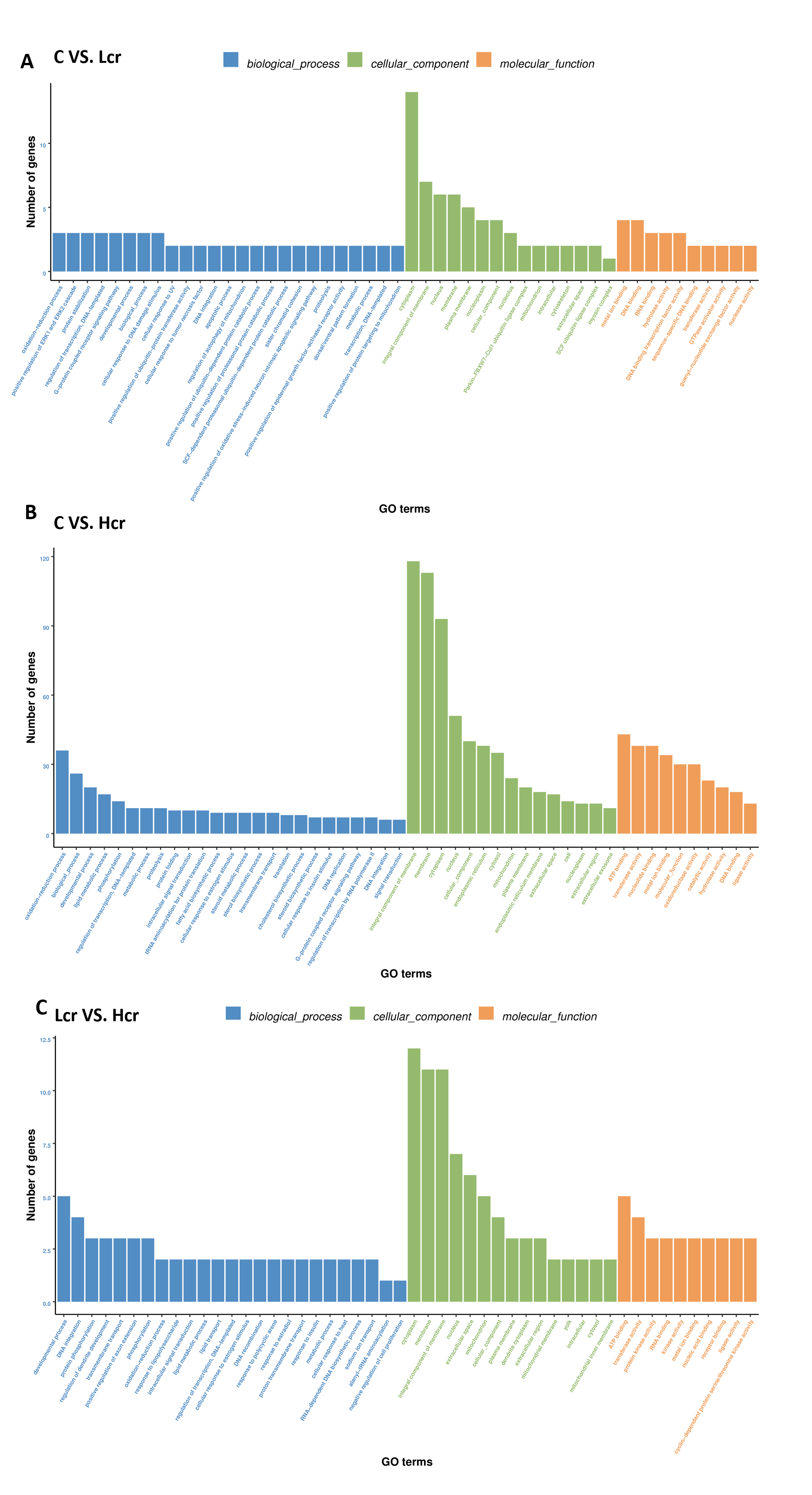

Supplement: Supplementary file 4 [file Image4.tif]
