# Supplementary material for: Chronic hexavalent chromium exposure induces oxidative stress-mediated molecular cascades in Thymallus grubii gills: evidence from integrated transcriptomics and metabolomics
Source: Front Immunol. 2025 Jul 9;16:1633174. doi: 10.3389/fimmu.2025.1633174 (PMC12285536; doi:10.3389/fimmu.2025.1633174)
Supplement: Supplementary file 5 [file Table1.docx]

Table. S1

Primers used in this study.

| Gene name | Gene |  | Nucleotide sequence (5–3′) |
| --- | --- | --- | --- |
| *Peroxisome Proliferator Activated Receptor γ* | *PPAR-γ* | Forward | GAGGGAGAGGCAACGTCAAA |
|  |  | Reverse | TCCCCGTCATCTCTCAGGTT |
| *Cyclooxygenase-2* | *COX-2* | Forward | TGTGGGCTTTGACATCCTCA |
|  |  | Reverse | TTCATGTCACTCAGGTGGGC |
| *interleukin 10* | *IL-10* | Forward | TATAGAGGGCTTCCCCGTCA |
|  |  | Reverse | CCATAGTGTGACACCCCACC |
| *transforming growth factor-β* | *TGF-β* | Forward | AGCAGGGCAACTCTGATGAC |
|  |  | Reverse | CTGGTGAATGATGGCCAGGT |
| *Nuclear factor κB* | *NF-κB* | Forward | AACGACCTCACAGCACAGTT |
|  |  | Reverse | TGATAGTGCTGCCCCCTTTG |
| *interleukin 8* | *IL-8* | Forward | GACCGAGAGCAAACGCATTG |
|  |  | Reverse | TGACCCTCTTGACCCAAGGA |
| *70-kDa heat shock protein* | *HSP70* | Forward | ATCGGATCGGTCATTCTGGC |
|  |  | Reverse | GGCGAGGACTTCGGTAGATG |
| *cysteinyl aspartate specific proteinase 9* | *Caspase-9* | Forward | CCAGGGCAACAGGAAGAGTT |
|  |  | Reverse | CCGTTGGCCGTGTATTCTGA |
| *cysteinyl aspartate specific proteinase 3* | *Caspase-3* | Forward | ACAGACACTCCGCCTCATTTG |
|  |  | Reverse | CAGTACCTCTGCAAGCCTGG |
| *Glutathione peroxidase* | *GPx4* | Forward | GTCAGGAACCTGGCACTGAA |
|  |  | Reverse | GCTCCTTCAGCCACTTCCAT |
| *TRINITY_DN6994_c0_g1* |  | Forward | ACTTGTTTCGTGGCTAGTGGA |
|  |  | Reverse | TGCCGCTGATACAATGTCCC |
| *TRINITY_DN540_c0_g2* |  | Forward | TGTCAGCAGGTCTACTTGGAG |
|  |  | Reverse | ATTCCACCCATGCGACTACT |
| *TRINITY_DN461_c0_g2* |  | Forward | GCTCTAGAAAGTTGGCAGAGGT |
|  |  | Reverse | GCGATAGGCACTGGGACTTT |
| *TRINITY_DN14148_c0_g1* |  | Forward | AGAGACACCGAAAGATACCTGG |
|  |  | Reverse | GCATGGTGAGGGGTTTAGGC |
| *TRINITY_DN25813_c1_g1* |  | Forward | CGCTATCTACCCATCTGCCC |
|  |  | Reverse | GCCCAGTCACTGTTCATGGT |
|  | β-actin | Forward | GCTCTGCCCCACGCCATCCT |
|  |  | Reverse | CGGTGCCCATCTCCTGCTCAAAG |
